# The impact of selected xanthophylls on oil hydrolysis by pancreatic lipase: in silico and in vitro studies

**DOI:** 10.1038/s41598-024-53312-9

**Published:** 2024-02-01

**Authors:** Grzegorz Dąbrowski, Sylwester Czaplicki, Marcin Szustak, Eliza Korkus, Edyta Gendaszewska-Darmach, Iwona Konopka

**Affiliations:** 1 Faculty of Food Sciences, Chair of Plant Food Chemistry and Processing, University of Warmia and Mazury in Olsztyn, Pl. Cieszyński 1, 10-726 Olsztyn, Poland; 2 Faculty of Biotechnology and Food Sciences, Institute of Molecular and Industrial Biotechnology, Lodz University of Technology, Stefanowskiego 2/22, 90-537 Lodz, Poland

**Keywords:** Enzyme mechanisms, Fats, Oils

## Abstract

Lipase inhibition is one of the directions to control obesity. In vitro assays have confirmed the inhibitory effect of selected xanthophylls, including astaxanthin, fucoxanthinol, fucoxanthin, and neoxanthin. Similarly, an in-silico study also demonstrated the successful inhibition of pancreatic lipase by astaxanthin. Unfortunately, the efficacy of these protocols in the emulsion state typical of lipid digestion remains untested. To address this issue, the current study employed the pH–stat test, which mimics lipid digestion in the gastrointestinal tract, to evaluate native and prepared sea buckthorn and rapeseed oils with varying xanthophyll contents from 0 to 1400 mg/kg oil. Furthermore, a molecular docking of zeaxanthin and violaxanthin (commonly found in plant-based foods), astaxanthin (widely distributed in foods of marine origin) and orlistat (approved as a drug) was performed. The in-silico studies revealed comparable inhibitory potential of all tested xanthophylls (variation from − 8.0 to − 9.3 kcal/mol), surpassing that of orlistat (− 6.5 kcal/mol). Nonetheless, when tested in an emulsified state, the results of pH–stat digestion failed to establish the inhibitory effect of xanthophylls in the digested oils. In fact, lipolysis of native xanthophyll-rich sea buckthorn oil was approximately 22% higher than that of the xanthophyll-low preparation. The key insight derived from this study is that the amphiphilic properties of xanthophylls during the digestion of xanthophyll-rich lipids/meals facilitate emulsion formation, which leads to enhanced fat lipolysis.

## Introduction

Obesity is a complex multifactorial medical condition that stems from excessive or abnormal body fat accumulation. This condition has far-reaching implications on health, which can be detrimental and potentially life-threatening^1^. Currently, six anti-obesity drugs are approved by the U.S. Food and Drug Administration for long-term use to combat obesity. These pharmacological agents include phentermine/topiramate, liraglutide, naltrexone/bupropion, semaglutide, setmelanotide, and orlistat^2^. Among the anti-obesity medications, only orlistat can inhibit pancreatic lipase activity. However, in recent years, there has been a growing interest in exploring the potential of herbal inhibitors of pancreatic lipase^3^. Among them, many natural food ingredients such as soy proteins, protamine, ovalbumin, chitosan, phytic acids, terpenes, phenolic compounds, saponins, etc., have been found to inhibit the digestion of triacylglycerols (TAGs)^4–7^. Similarly, several studies have shown the inhibitory effect of certain carotenoids on pancreatic lipase. This was confirmed in a spectrometric assay using 4-nitrophenyl butyrate for *Phaffia rhodozyma* astaxanthin, which reduced lipase activity through a non-competitive manner within the tested ranges of 0–70 µg/mL^8^. Astaxanthin at a dose of 40–50 µg/mL was equivalent to the inhibitory effect of 0.6 µg orlistat per mL. This study demonstrated that astaxanthin changed the secondary conformation of lipase and thus reduced enzyme activity. Similarly, Matsumoto et al.^9^ showed that two marine carotenoids (fucoxanthin and fucoxanthinol) inhibited lipase activity in the gastrointestinal lumen and suppressed lipid absorption in a rat model. In this study, the total amounts of TAGs released into the lymph 4 h after ingestion were 53.1, 59.4, and 113.5 µmol for the fucoxanthinol- and fucoxanthin-fed (2 mg) and carotenoid-free rat groups, respectively. An in vitro triolein digestion experiment was also performed in this study. The inhibition of pancreatic lipase was dose-dependent in the range of 0–1 mg/mL; with a 50% lipase inhibition (IC_50_) by ca. 500 µg/mL of both carotenoids. The freshwater algae *Oedogonium intermedium* extract, which is rich in neoxanthin, also showed pancreatic lipase inhibitory activity in a fluorometric assay with 4-methylumbelliferyl oleate^10^. For this extract, IC_50_ of approx. 4 and 1 mg/mL was determined for crude and saponified lipid extracts, respectively. Hitoe and Shimoda^11^ investigated the effect of fucoxanthin (1 or 3 mg daily) in a *double-blind placebo-controlled* study. Capsules containing fucoxanthin or placebo were administered to male and female Japanese adults with a body mass index of more than 25 kg/m^2^ for four weeks. Relative parameters of body condition were significantly better in the fucoxanthin-treated groups than in the placebo group.

As shown, the majority of the studies conducted to date have utilized only buffer solutions in spectrophotometric or fluorometric assays using *p*-nitrophenyl-palmitate (*p*-NPP) or -butyrate (*p*-NPB)^8,12^ or 4-methylumbelliferyl oleate^13^ without the effect of emulsification observed during lipid digestion in the human gastrointestinal tract. Although Matsumoto et al.^9^ used an in vitro test with triolein as a substrate, the emulsion was prepared from triolein with taurocholate (bile acid) and buffered saline (HEPES). In the authors’ opinion, the tested xanthophylls (fucoxanthin or fucoxanthinol) should be added to the mixture before emulsification to create an emulsion that mimics the actual state of digestion.

All the carotenoids mentioned above, whose inhibitory effect on pancreatic lipase has been experimentally confirmed, are xanthophylls. The main source of these xanthophylls is seafood, which is often avoided by consumers due to its unpleasant fishy smell and taste^14^. Among the plant sources, the oil from the pulp of sea buckthorn is extremely abundant in carotenoids, reaching levels of up to 6300 mg/kg^15^. In the ripe fruits of this plant, xanthophylls such as lutein, zeaxanthin, and β-cryptoxanthin account for approx. 40–60% of the total carotenoids^16,17^. Tkacz et al.^18^ demonstrated that 70% acetone extracts from six sea buckthorn fruits had anti-lipase activity using a *p*-nitrophenyl acetate assay (IC_50_ ranged from 4.19 to 14.02 mg/mL).

In general, the inhibition of pancreatic lipase is related to the blockage of the entrance channel of the catalytic site of this enzyme at Ser152, Asp176, and His263^8^. The most significant residue for lipolytic activity is Ser152. Activation of the enzyme occurs at the water–oil interfaces^5^. Based on in-silico molecular docking, Ahmed et al.^19^ found that out of 3770 compounds tested, pancreatic lipase was mainly inhibited by 10 molecules found in plants: kushenol K (flavonoid from *Sophora flavescens*), rosmarinic acid (phenolic compound—ester of caffeic acid and 3,4-dihydroxyphenyllactic acid found in *Perilla frutescens*, the genus *Salvia*, *Salvia officinalis, Mentha spicata*), reserpic acid (yohimban alkaloid found in *Rauvolfia vomitoria*), munjistin (1,3-dihydroxyanthraquinone-2-carboxylic acid from *Rubia argyi, Rubia yunnanensis*), leachianone G (tetrahydroxyflavanone from *Morus alba, Sophora flavescens, and Lespedeza cyrtobotrya*), cephamycin C (β-lactam antibiotic produced by *Streptomyces clavuligerus*, *S. cattleya* and *Nocardia lactamdurans*), arctigenin (lignan found in *Arctium lappa* and *Saussurea heteromalla*), 3-*O*-acetylpadmatin (flavonoid from *Dittrichia graveolens* and *Chrysothamnus viscidiflorus*), geniposide (genipin glycoside found in *Gardenia jasminoides*) and obtusin (dihydroxyanthraquinone found in *Laurencia obtuse* and *Senna obtusifolia*). For these compounds, the docking scores were between − 12.03 and − 13.78 kcal/mol, and thus higher than for orlistat (whose value was estimated as − 10.20 kcal/mol in the cited study). It is important to note that these compounds belong to polyphenolics, alkaloids, β-lactam antibiotics, and anthraquinones, and none of them belong to the xanthophylls group.

The presented analysis of the current state of knowledge indicates an inconsistency in the evaluation of the influence of xanthophylls on the activity of pancreatic lipase. The main objective of the study is therefore:

1. to demonstrate the ability of selected xanthophylls (astaxanthin typical for marine foods vs. zeaxanthin and violaxanthin typical for plant foods) to inhibit the pancreatic lipase activity by molecular docking (in silico study),
2. to compare these results with the results of free fatty acid release during digestion of different lipid matrices (xanthophyll-poor or -rich) in the pH–stat test.

## Results and discussion

### Basic molecular properties and predicted drug-like activity of selected xanthophylls (zeaxanthin, violaxanthin, astaxanthin), orlistat, and ten natural compounds considered to be the best inhibitors of pancreatic lipase^19^

Table 1 shows the physical properties and predicted bioactivity of the confirmed pancreatic lipase inhibitors^19^ and three xanthophylls and orlistat used in the current study. The properties were calculated using the “Molinspiration Cheminformatics” tool. ^20^. According to the service, the compounds listed are composed of 21–44 atoms and have TPSA values ranging from 40.46 (zeaxanthin- predominantly hydrophobic) to 211.59 (cephamycin C-predominantly hydrophilic). The analyzed compounds also differed in miLogP values, which affected their oral/intestinal absorption^21^. The molecular weights and volumes of these compounds were less variable, reaching values from 284.22 to 600.88, and from 225.61 to 615.52 for munjistin and violaxanthin, respectively. This shows that pancreatic lipase inhibitors have variable molecular properties. Among the compounds tested, the properties of orlistat are most similar to those of the three xanthophylls used. Some of these compounds are enzyme inhibitors.

**Table 1:** Selected characteristics of compounds that can inhibit pancreatic lipase (based on Molinspiration^20^).

Common name	N atoms	miLogP	TPSA	MW	Vol	Predicted activities
Kushenol K	34	4.04	136.68	472.53	433.02	GPCRL, NRL, PIn, EIn
Rosmarinic acid	26	1.63	144.52	360.32	303.54	NRL, EIn
Reserpic acid	29	2.33	95.02	400.48	364.37	GPCRL, ICM, PIn, EIn
Munjistin	21	2.84	111.90	284.22	225.61	EIn
Leachianone G	26	4.28	107.22	356.37	315.62	GPCRL, NRL, EIn
Cephamycin C	30	-3.98	211.59	446.44	363.91	PIn, EIn
Arctigenin	27	2.22	74.23	372.42	341.29	GPCRL, EIn
3-O-acetylpadmatin	26	1.95	122.53	360.32	300.36	NRL
Geniposide	27	-1.53	155.15	388.37	331.54	GPCRL, EIn
Obtusin	25	3.32	102.30	344.32	291.81	–
Orlistat	35	9.01	81.71	495.75	525.13	GPCRL, PIn, EIn
Astaxanthin	44	8.60	74.60	596.85	612.41	–
Zeaxanthin	42	9.39	40.46	568.89	608.05	NRL
Violaxanthin	44	8.99	65.51	600.88	615.52	–

Natoms, number of atoms; miLogP, octanol/water partition coefficient; TPSA, topological polar surface area; MW, molecular weight; Vol, molecular volume; GPCRL, GPCR ligand; ICM, ion channel modulator; KIn, kinase inhibitor; NRL, nuclear receptor ligand; PIn, protease inhibitor; EIn, enzyme inhibitor.

### In silico study of the inhibitory effect of different xanthophylls against pancreatic lipase by molecular docking

**Figure 1:**
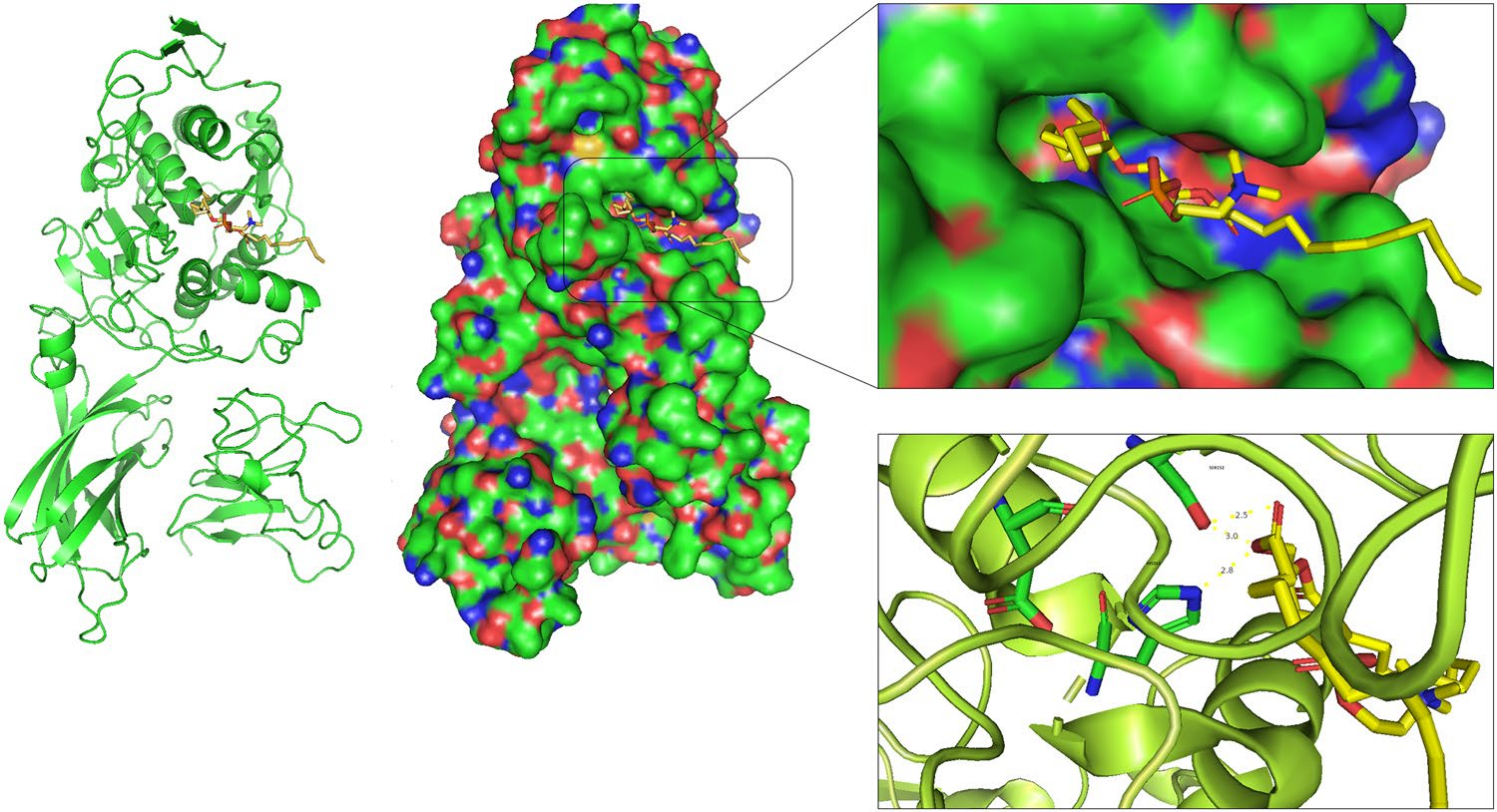
Docking simulation of human pancreatic lipase with diundecyl phosphatidylcholine (PDB: 1LPA).

Molecular docking is a theoretical simulation based on bioinformatics that studies the interaction between molecules (such as ligands and receptors) and predicts their binding modes and affinity via a computer platform^22^. This technique was employed to gain a theoretical insight into the interaction between the pancreatic lipase and three xanthophyll ligands. The crystal structure of the complex of the human pancreatic lipase with diundecyl phosphatidylcholine (PDB: 1LPA) was used as a template for the docking simulation (Fig. 1).

**Figure 2:**
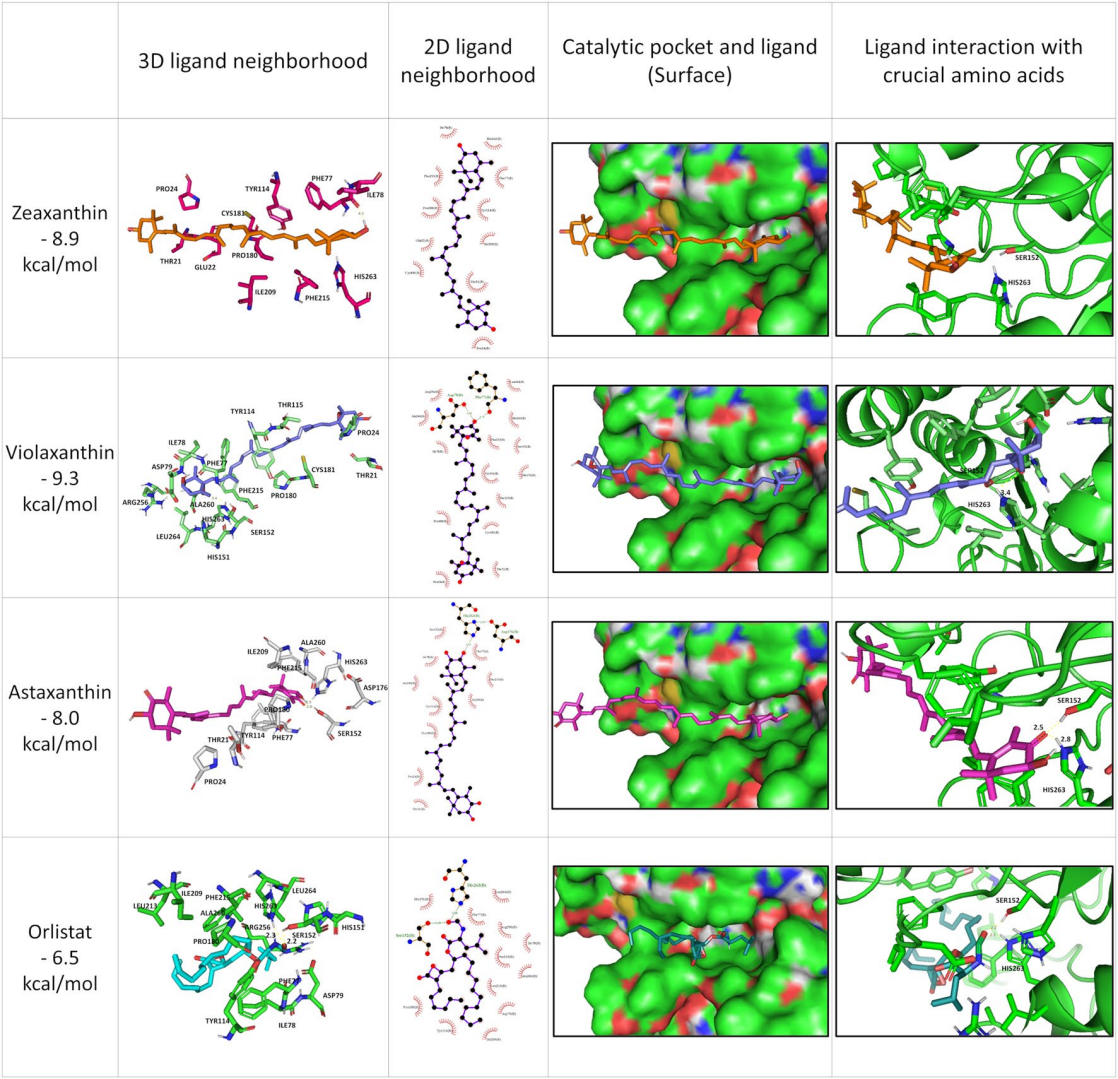
Docking simulation of human pancreatic lipase with zeaxanthin, violaxanthin, astaxanthin, and orlistat.

Diundecyl phosphatidylcholine creates hydrogen bonds between crucial amino acids located in the catalytic center of lipase (Ser152, His265) and oxygen atoms of the ester bond. By molecular docking, zeaxanthin, violaxanthin, and astaxanthin were localized in the active site of pancreatic lipase with the affinity of − 8.9, − 9.3, and − 8.0 kcal/mol, respectively. The primary amino acid residues responsible for the ligand–protein interaction (found in at least two models) were Thr21, Pro24, Phe77, Ile78, Tyr114, Ser152, Pro180, Ile209, Phe215, Ala260, and His2. Moreover, hydrogen bonds were only found between astaxanthin and Ser152 (2.5 Å) and His263 (2.8 Å). The distance between violaxanthin and His263 (3.4 Å) was too large to form a hydrogen bond. Otherwise, LigPlus + showed an additional interaction between violaxanthin and Asp79 and the peptide chain of Phe77. There was no trace of hydrogen bonds between zeaxanthin and amino acids in the enzyme pocket. Nevertheless, zeaxanthin fills the entire catalytic pocket through many other interactions (hydrophobic, van der Walls or other interactions between ligand molecule and pancreatic lipase molecule), resulting in a high affinity. Figure 2 shows that all investigated ligands occupy the entire space in the catalytic site, which probably leads to a blocking of the activity of this enzyme. Additionally, orlistat was docked to the enzyme under the same conditions. This compound formed hydrogen bonds with Ser152 (2.2 Å) and His263 (2.3 Å) (Fig. 2).

Previous docking studies have shown that pancreatic lipase can be inhibited by yeast, algae, and chemically produced astaxanthins with a binding energy of − 4.84 to − 5.71 kcal/mol, similar to *p*-NPB with a value of − 4.30 kcal/mol^8^. Similarly, Shamarao and Chethankumar^23^ synthesized and characterized the total lutein oxidized products (LOPs) by exposing lutein to direct sunlight. The total LOPs showed significant inhibition of pancreatic lipase activity with an IC_50_ of 1.6953 μg/mL. The most active compound—LOP6 [4-((1E,3E,5E)-3,7-dimethylocta-1,3,5,7-tetraen-1-yl)-3,5,5-trimethylcyclohex-3-enol] with an approximate molecular mass of 274.25 showed a binding energy of − 5.35 kcal/mol for pancreatic lipase.

Thus, this suggests that the interaction between pancreatic lipase and xanthophylls used could inhibit lipase activity as successfully as orlistat does. Although the results of the present study clearly indicate that astaxanthin can form hydrogen bonds and could be a competitive inhibitor, the computational models of pancreatic lipase – astaxanthin and *p-*NPB presented by Du et al.^8^ showed lower values for this activity*.* The different origin of the enzyme* (Cavia porcellus* in the cited study vs. *Homo sapiens* in the current study) is probably the reason for the different results.

### Release of fatty acids from xanthophyll-rich and xanthophyll-poor oil matrices

**Figure 3:**
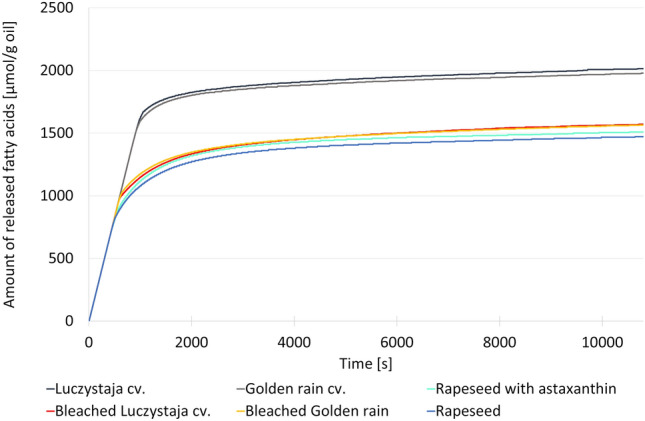
The kinetics of fatty acid release from prepared oils obtained by the in vitro method using the pH–stat protocol.

The xanthophyll-rich oils tested contained 402, 352, and 1400 mg of these compounds per kg, whereas they were below the detection limit in the xanthophyll-poor oils (Table 2). The kinetics of lipolysis of the studied oils were monitored by the in vitro method using the pH–stat protocol. The results of these experiments are presented in Fig. 3.

**Table 2:** Carotenoid contents (mg/kg oil), liberated free fatty acids (FFA) content, and yield of lipolysis of the tested oils.

	Oils rich in xanthophylls			Oils poor in xanthophylls		
	Luczystaja cv. native	Golden rain cv. native	Rapeseed with astaxanthin	Luczystaja cv. bleached	Golden rain cv. bleached	Rapeseed native
Lutein	37.4 ± 0.7^c^	34.7 ± 0.5^b^	< LOD^a^	< LOD^a^	< LOD^a^	< LOD^a^
Zeaxanthin	41.2 ± 0.6^c^	38.1 ± 0.0^b^	< LOD^a^	< LOD^a^	< LOD^a^	< LOD^a^
Zeinoxanthin or α-cryptoxanthin	41.3 ± 1.2^c^	36.6 ± 0.9^b^	< LOD^a^	< LOD^a^	< LOD^a^	< LOD^a^
β-Cryptoxanthin	93.1 ± 3.2^c^	69.0 ± 2.3^b^	< LOD^a^	< LOD^a^	< LOD^a^	< LOD^a^
Other xanthophylls	189 ± 5^b^	192 ± 49^b^	< LOD^a^	< LOD^a^	< LOD^a^	< LOD^a^
Astaxanthin	< LOD	< LOD	1400 ± 0	< LOD	< LOD	< LOD
Total xanthophylls	402 ± 11^b^	352 ± 73^b^	1400 ± 0^c^	< LOD^a^	< LOD^a^	< LOD^a^
Total carotenoids	671 ± 18^b^	1418 ± 11^c^	1400 ± 0^c^	< LOD^a^	< LOD^a^	10.2 ± 0.3^a^
µmol of released FFA/g of oil	2015 ± 46^c^	1978 ± 36^c^	1510 ± 20^ab^	1571 ± 24^b^	1564 ± 60^ab^	1468 ± 30^a^
g of released FFA/g of oil	0.516 ± 0.01^b^	0.506 ± 0.009^b^	0.426 ± 0.006^a^	0.402 ± 0.006^a^	0.400 ± 0.015^a^	0.414 ± 0.008^a^
Yield of lipolysis (%)	57.3 ± 1.3^b^	56.3 ± 1.0^b^	47.3 ± 0.6^a^	44.7 ± 0.7^a^	44.5 ± 1.7^a^	46.0 ± 0.9^a^

Data in the row marked with the same letter are not statistically different; LOD, limit of detection.

After 3 h of lipolysis, up to 2015, 1978 and 1510 µmol/g fatty acids were released from native sea buckthorn oils (Luczystaja cv. and Golden rain cv.), and rapeseed oil supplemented with 1400 mg astaxanthin per kg oil, respectively (Table 2). Surprisingly, significantly lower amounts of liberated free fatty acids were found in the bleached oils from both sea buckthorn cultivars, with similar final values (1564–1571 µmol/g) and in the native rapeseed oil with 1468 µmol/g. This indicates that the yield of lipolysis in bleached oils was only approx. 78% of the yield in native oils, while the difference in rapeseed oil was significantly lower, but still about 3% more fatty acids were released from the astaxanthin- supplemented oil. Considering the molar mass of palmitic and oleic acid as the main representatives of fatty acids in sea buckthorn and rapeseed oil fatty acids (approx. 256 and 282 g per mol, respectively), the final mass yield of lipolysis was determined to be approx. 57% in both native sea buckthorn oils and only approx. 45% in both bleached oils. In the rapeseed oils, variation was negligible. The fastest hydrolysis rate was evaluated for all oils in the first twenty minutes and was responsible for approx. 80% of all FFA liberated during the 3-h lipolysis.

The results of this part of the experiment did not confirm the inhibitory effect of total xanthophylls detected in sea buckthorn oils (402 and 352 mg/kg in Luczystaja, and Golden rain cvs., respectively) and astaxanthin applied to refined rapeseed oil. In contrast, the increased lipolysis of xanthophyll-rich/supplemented oils can be explained by their chemical properties. Together with carotenes, xanthophylls belong to tetraterpenoids but differ from them by the presence of oxygen atoms in the molecules in methoxy, hydroxy, keto, carboxy, and epoxy groups^24^. Xanthophylls exhibit both lipophilic and hydrophilic properties. In an ingested meal containing lipid emulsions (natural oil bodies or the resulting digested liquid), these compounds should occupy a transmembrane location, stabilized by the formation of hydrogen bonds between the terminal hydroxyl groups of xanthophylls and the opposite polar regions of the lipid layer^25^. According to Shibata et al.^26^ astaxanthin, as a representative of this group, has a polar group at the end of the molecule. It is a typical amphiphilic molecule and forms a stable monolayer of droplets, which is essential for the formation of an O/W emulsion. This indicates that only a polar group of astaxanthin (hydroxyl and ketone groups) can be deposited on the surface of an oil droplet and interact with the active site of the lipase. The activity of most lipases is located at the lipid–water interfaces enabled by a mobile lid domain located over the active site. A lid is responsible for the catalytic activity as it protects the active site of the enzyme molecule. In pure aqueous media, the lid is predominantly closed, whereas the presence of a hydrophobic layer leads to a partial opening. Therefore, the lid controls the enzyme activity^27^. The difference in the lipolysis yield between native sea buckthorn oils and astaxanthin-enriched rapeseed oil can also be influenced by other factors. For example, the position of the fatty acid in the glycerol backbone, the chain length of the fatty acid and its degree of unsaturation are the key factors that affect the activity of lipases^28^. For sea buckthorn oils, the chain length seems to be decisive. The cited authors found that various lipase activities were much higher in tributyrin than in food-grade oils or pure TAGs with long-chain fatty acids (such as triolein or tripalmitin). A similar phenomenon was reported by Ye et al.^29^. They conducted a 240-min in vitro digestion assay and found that the release rate of shorter-chain saturated fatty acids (C16:0 in palm oil) was higher than that of longer-chain polyunsaturated fatty acids (C18:3 n-3 in linseed oil).

The obtained results seem to contradict the molecular docking data. However, the extent of fat emulsification could be an important factor influencing the activity of digestive lipases and regulating the digestion of dietary fats. Since the lipolysis reaction depends on the availability of the oil–water interface, the area of the droplet interface has a significant influence on the degree of hydrolysis^4^. When emulsion droplets flocculate and coalesce, and the lipid surface area available for lipase binding is reduced, the rate of TAG hydrolysis is expected to be reduced^4^. A smaller initial lipid droplet size, resulting in a larger surface area, improves fat digestion. The degree of TAG hydrolysis can range between 5 and 37% depending on the size of lipid droplets^30^. The size of the emulsion droplets and the nature of the interfacial layer during food preparation vary greatly. The scale of the droplet size can be measured in both the nano- and micrometer range, and the interface is often stabilized by proteins and/or phospholipids, but more complex interfaces containing solid particles and biopolymer multilayers are also possible^4^.

**Figure 4:**
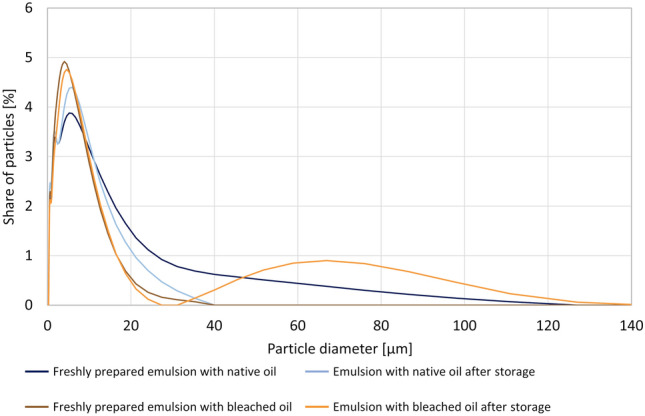
Particle size distribution of O/W emulsions prepared with sea buckthorn native and bleached oils.

Since the extent of lipolysis could be related to the particle size of the emulsion, the droplet size of the dispersed lipid phase was measured by laser diffraction. In freshly prepared emulsions, the median size of the droplets in native and bleached sea buckthorn oils was comparable, with a diameter of approx. 6 µm (Fig. 4). The distribution of O/W droplets in both emulsions was significantly different after the storage of the emulsion. In the bleached oil, the proportion of large droplets with diameters of 30–130 µm increased visibly. This indicates degradation of the emulsion, which eventually reduces the extent of lipolysis. It seems that the almost complete reduction of amphiphilic compounds in bleached oils (chlorophylls, pheophytins, carotenoids, free fatty acids, MAGs, DAGs, xanthophylls, etc.) diminished the possibility of the formation of a stable emulsion^31^.

In an additional experiment on lipolysis using a monolayer protocol (data not shown as additional table or figure), bleached or astaxanthin-supplemented Golden rain oils were carefully added to a lipase solution in PBS buffer. The mixture was mildly shaken at 37 °C to prevent emulsion formation. After 3 h of digestion in bleached oil, liberation of approx. 206 µmol of free fatty acids per 1 g of oil was determined, while in oil supplemented with astaxanthin, there was only about 138 µmol of free fatty acids released per 1 g of oil. The obtained differences are statistically significant (*p* ≤ 0.05) and confirm the inhibitory effect of astaxanthin against pancreatic lipase in a non-emulsion environment. However, lipid digestion without emulsion creation is not typical for lipolysis in the human gastrointestinal tract. Finally, it was also concluded that in typical food emulsions, the amphiphilic properties of xanthophylls are more important than their inhibitory effect on pancreatic lipase.

## Conclusions

The present study investigated the potential of three xanthophylls, namely astaxanthin, violaxanthin, and zeaxanthin, to inhibit pancreatic lipase. A docking experiment was performed to create a ligand-enzyme complex, which yielded a reduced energy number density of 8.0–9.3 kcal/mol, which confirmed the possibility of inhibiting the *Homo sapiens* lipase. The inhibitory effect of astaxanthin as the representative xanthophyll was also verified in a newly developed experiment protocol with a stationary/monolayer model of lipids digestion. However, in a typical emulsion model for in vitro digestion, an approx. 20% lower yield of lipolysis was observed for bleached, carotenoids-free oils. This result was attributed to changes in the size of emulsion droplets and the diminished content of surface-active compounds caused by the bleaching stage. The study concludes that lipolysis is favored by the native sea buckthorn oils xanthophylls. In summary, the present research provides valuable insights into the potential of xanthophylls in inhibiting the *Homo sapiens* pancreatic lipase and highlights the importance of considering the experimental protocol for investigating the effect of xanthophylls on lipolysis.

## Methods

### In silico analysis of molecular properties and possible drug-like activities

This analysis was performed based on canonical SMILES structure^32^ for ten compounds with the greatest inhibitory activity against pancreatic lipase^19^ as well as for zeaxanthin, violaxanthin, astaxanthin, and orlistat^20^.

The Molinspiration service provides comprehensive insights into the molecule’s hydrophobicity, electronic distribution, hydrogen bonding characteristics, size, and flexibility and also the presence of various pharmacophoric features influencing the behavior in a living organism, including, e.g., bioavailability, transport properties, affinity to proteins, reactivity, toxicity, or metabolic stability^20^. In the current study, this calculator was used to determine such properties as Natoms—number of atoms; miLogP—octanol/water partition coefficient; TPSA—topological polar surface area; MW—molecular weight; Vol—molecular volume; GPCRL—GPCR ligand activity; ICM—ion channel modulator activity; KIn—kinase inhibitor activity; NRL—nuclear receptor ligand activity; PIn—protease inhibitor activity; EIn—enzyme inhibitor activity.

### In silico molecular docking of pancreatic lipase with zeaxanthin, violaxanthin, astaxanthin, and orlistat

Molecular docking was used to compare the binding energies of three xanthophylls and orlistat, a well-known lipase inhibitor. Docking of xanthophylls to the human pancreatic lipase was performed using AudoDockVina 1.2.0 software. The structures of carotenoids and orlistat were obtained from the PubChem database (zeaxanthin CID: 5,280,899; violaxanthin CID: 448,438; astaxanthin CID: 5,281,224; orlistat CID: 3,034,010). The human pancreatic lipase structures were downloaded from the Protein Data Bank (ID: 1LPA). Ligand models were converted to .mol2 files via Open Babel GUI 2.4.0 software and subsequently prepared for the docking software with AutoDockTools 1.5.7 to an acceptable .pdbqt file for AutoDockVina. Hydrogen atoms and polarity were added to proteins with AutoDockTools 1.5.7 and converted to a .pdbqt file. Each ligand was docked in a grid box (40 A × 40 A × 40 A) centered at (5.138, 28.79, 62.438) with exhaustiveness set to 20. During stimulation, the ligands were considered fully flexible, while the lipase was kept rigid. Two different programs were used to present the binding site. PyMOL software was used to visualize a 3D model with hydrogen interaction. LigPlus + software was used to predict all interaction ligand–protein in 2D images.

### Materials for in vitro experiments of lipolysis

Six distinct oils were involved in this study, with three classified as rich in xanthophylls and the remaining three considered poor in this particular compound.

Oils abundant in xanthophylls:

1. Sea buckthorn flesh oil of Luczystaja cv.,
2. Sea buckthorn flesh oil of Golden Rain cv.,
3. Commercial refined rapeseed oil supplemented by astaxanthin (1400 mg/kg of oil),

Oils poor in xanthophylls:

4. Bleached Luczystaja cv. flesh oil,
5. Bleached Golden Rain cv. flesh oil,
6. Commercial refined rapeseed oil.

The methodology for extracting flesh oils from two distinct cultivars of sea buckthorn was replicated from the authors’ previous study^15^. The bleaching procedure was adopted from Korkus et al.^33^. Briefly, a mixture comprising 2.5 g of oil, 15 ml of hexane, and 5 g of active carbon was mixed in a Multi RS-60 Rotator (Biosan, Riga, Latvia) at room temperature for 20 min, rotating at 30 rpm. The mixture was then centrifuged in a 5910R centrifuge (Eppendorf, Hamburg, Germany) for 10 min at a relative centrifugal force of 4816×*g*. The bleached oil was obtained by separating the carbon by filtration and evaporating the hexane using a rotary vacuum evaporator (Büchi, Flawil, Switzerland) at 45 °C. Commercially refined rapeseed oil was bought on the local market in Olsztyn (Poland), while astaxanthin was from Merck (Darmstadt, Germany).

### In vitro digestion of studied oils in emulsion model

The oils prepared according to the previous point were digested in the uniform procedure. Briefly, 1 g of oil, 10 ml of bile salt in PBS solution (0.188 g/10 ml), 2 ml of CaCl_2_ in water (0.551 g/20 ml), and 47.4 ml of NaCl in PBS (1.338 g/100 ml) were mixed and 0.1 M NaOH or 0.1 M HCl solution were used to adjust pH of the obtained mixture to 8.5. Subsequently, a T25 Ultra Turrax (IKA-Werke, Staufen, Germany) homogenizer was used to prepare the emulsion that was then hydrolyzed in a 907 Titrando titrating device (Metrohm, Herisau, Switzerland). To initiate lipid hydrolysis, 0.18 g of lipase (Merck, Darmstadt, Germany; 100–650 U/mg of protein in olive oil model) in PBS buffer (pH = 8.5) was added to 30 mL of the homogenate. The pH of the mixture was maintained at a constant level using 0.1 M water solutions of NaOH or HCl. The total volume of 0.1 M NaOH used to neutralize free fatty acids liberated during digestion was determined. Additionally, the kinetics of the reaction were measured, and hydrolysis was carried out over a period of 3 h. The results, which represent the average of at least two separate determinations, are presented as µmol and g of liberated fatty acids per oil mass. To simplify, the calculation of the yield of liberated FFA from sea buckthorn oils and rapeseed oils, palmitic acid and oleic acid used as standards, with molecular weights of 256 g/mol and 282 g/mol. It was assumed that the used oils were composed of 100% TAGs, with approximately 10% of glycerol share in TAGs. The final yield of lipolysis was then calculated based on this information.

### In vitro digestion of studied oils in a monolayer model

0.18 g of the lipase in PBS buffer (pH = 8.5) was placed in a 100 ml glass beaker. Next, 0.5 g of either native rapeseed oil or oil supplemented with 1,400 mg of astaxanthin per kg of oil was carefully applied to the lipase solution to prevent lipid droplet formation. The solution was stirred at a mild (60 rpm) rate in a shaking incubator (Incu-Shaker Mini, Benchmark Scientific, Sayreville, NJ, USA) for 3 h at 37 °C. After this time, liberated fatty acids were neutralized by 0.1 M NaOH in a Titrando device. A control oil sample (without lipase in PBS buffer) was used to calculate the liberated fatty acids from both oils. Results are the average of two separate determinations and are presented as µmol and g of liberated fatty acids per oil mass.

### Oil droplet size and microstructural characteristic of O/W emulsions (results presented for Golden rain cv. oils, only)

In the resultant emulsions of native and bleached oils, the sizes of dispersed O/W phase particles were determined by laser diffraction analysis using Mastersizer 2000 (Malvern Panalytical, Worcestershire, United Kingdom).

### Carotenoid composition of oils

Carotenoids were analyzed with an RP-HPLC technique according to Czaplicki et al.^34^. The oil sample was diluted in hexane containing β-Apo-8'-carotenal as an internal standard and saponified with 6 ml of 40% methanolic KOH solution in a Multi RS-60 Rotator (Biosan, Riga, Latvia) at room temperature in the dark for 16 h. The solution was then supplemented with 30 mL of hexane and adjusted to 50 mL with 10% Na_2_SO_4_. The lower phase was separated, triple-rinsed with 10 ml of hexane, and collected with the upper organic phase. The organic solvent was evaporated at 40 °C under a nitrogen stream and dissolved in 2 ml of a methanol: dichloromethane (45:55 v/v) solution. The chromatographic analysis of carotenoids was conducted according to the modified Emenhiser et al. (1995) method. Briefly, the analysis was carried out using a 1200 series liquid chromatograph manufactured by Agilent Technologies (Palo Alto, CA, USA), equipped with a diode array detector (DAD) from the same manufacturer. Separation was performed at 30 °C on a YMC-C30 250 × 4.6 mm, 5 µm column and YMC-C30 10 × 4.6 mm, 3 µm precolumn (YMC-Europe GmbH, Germany). A methanol/methyl tert-butyl ether (MTBE) gradient was programmed as in the Czaplicki et al.^34^ study. The absorbance was measured at the wavelength of 450 nm. Carotenoids were identified based on retention times of available standards (Sigma-Aldrich, USA) and by comparing the UV–Visible absorption spectra.

### Statistical analysis

The analyses were performed at least in duplicate, and the results obtained were analyzed using Statistica 12.5 software (TIBCO, Palo Alto, CA, USA). Analysis of variance (ANOVA) with the Duncan test for homogenous groups (*p* ≤ 0.05) was used for the determination of the differences between the samples.

## Data Availability

The datasets used and/or analysed during the current study available from the corresponding author on reasonable request.
